# Prevalence of Diabetes, Prediabetes, and Associated Factors in an Adult Chinese Population: Baseline of a Prediabetes Cohort Study

**DOI:** 10.1155/2020/8892176

**Published:** 2020-11-24

**Authors:** Xinjie Yu, Fang Duan, Da Lin, Hai Li, Jian Zhang, Qiuyu Wang, Xianglong Wang, Qian Zhao, Jiayu Deng, Guangwei Song, Qingqing Ji, Haihua Zheng, Xiang Chen, Guoyi Zhou

**Affiliations:** ^1^Affiliated Yueqing Hospital, Wenzhou Medical University, Wenzhou, Zhejiang, 325600, China; ^2^Zhongshan Ophthalmic Center, State Key Laboratory of Ophthalmology, Sun Yat-sen University, Guangzhou 510060, China; ^3^The Second Affiliated Hospital and Yuying Children's Hospital of Wenzhou Medical University, Wenzhou 325027, China; ^4^Department of Endocrinology, The First Affiliated Hospital of Sun Yat-sen University, Guangzhou, 510060, China

## Abstract

**Purpose:**

To report baseline information of a prediabetes mellitus (PDM) cohort with the aim of exploring related factors for the progression of PDM and its complications.

**Methods:**

This study is an exploratory and cross-sectional analysis of the baseline data from a cohort study. Residents aged 18 to 70 years from Houtang Village, Nanyue Town, Yueqing City, Zhejiang Province, China, were invited to participate between October 1, 2018 and July 1, 2019. Blood samples were collected for analysis, and questionnaire interviews were conducted to assess behavioral characteristics. The study participants were divided into DM, PDM, and normal groups for comparisons based on their blood work, and multiple multinomial logistic regression analyses were used to assess the risk factors for DM and PDM.

**Results:**

Data from 406 participants were used in the baseline analysis, with a mean age of 51.2 ± 11.0 years and 160 (33.0%) males. The number of participants in the DM, PDM, and normal group was 58 (14.3%), 166 (40.9%), and 182 (44.8%), respectively. The prevalence of DM was 14.3%, and the prevalence of PDM was 40.9%. The regression analysis showed that older age (relative risk ratio (RRR) = 1.06; 95% CI, 1.01–1.11, *P* = 0.018), higher systolic blood pressure (RRR = 1.04; 95% CI, 1.004–1.08, *P* = 0.030), higher BMI (RRR = 1.20; 95% CI, 1.06–3.06, *P* = 0.004), higher TG (RRR = 1.80; 95% CI, 1.06–3.06, *P* = 0.029), and higher WBC count (RRR = 1.32; 95% CI, 1.07–1.64, *P* = 0.010) were significantly associated with a higher risk of DM. Meanwhile, higher systolic blood pressure (RRR = 1.03; 95% CI, 1.004–1.06, *P* = 0.025) was the only factor significantly associated with a higher risk of PDM.

**Conclusion:**

The prevalence of DM and PDM is relatively high in this wealthy East China village population. Many modifiable risk factors exist for DM and PDM, which will be closely monitored during our longitudinal observation.

## 1. Introduction

Diabetes mellitus (DM) is one of the common noncommunicable diseases worldwide and affects over 451 million people, and this number is projected to rise to 693 million by 2045 [1]. Subjects with DM have an increased risk of developing other systemic diseases, as well as a higher disability and mortality risk. The prevalence of DM is rapidly increasing with the aging population and modern lifestyle, posing a great burden on individual well-being and socioeconomic development [2].

Prediabetes mellitus (PDM), referred to as intermediate hyperglycemia or borderline diabetes, represents the intermediate stage of abnormal glucose metabolism [3, 4]. It is estimated that by 2030, more than 470 million individuals will be prediabetic [5]. Twenty-five percent of individuals with PDM will progress to type 2 DM in 3 to 5 years, and 70% will eventually progress to DM during their life course [6]. The importance of PDM has been increasingly recognized, as a better understanding of PDM could help by allowing earlier identification of high-risk populations for intervention, thereby lowering the number of people with DM in the future.

Many environmental factors, including obesity and aging, have been reported to contribute to the DM epidemic [2]. Knowledge of the risk factors could aid in a better understanding of disease etiology and the development of effective preventive measures. The literature also indicates that lifestyle modifications for prediabetic patients can help reduce or prevent the progression of DM by 40–70% [7]. Thus, the identification of factors associated with DM and implementing interventions to tackle them are crucial for reducing the disease burden and improving health outcomes.

China has the world's largest diabetes population; however, the current data on DM and PDM epidemiology are limited, especially because most previous studies on diabetic complications are cross-sectional in nature. Recent studies have demonstrated that diabetic microvascular complications could begin in PDM patients [8, 9]. It is our interest to observe a group of healthy residents over a 10-year period to monitor the longitudinal transition from healthy subjects to PDM and ultimately DM with related risk factors. Here, we report our baseline data.

## 2. Methods

### 2.1. Study Population and Sampling Methods

This study included baseline data from a longitudinal PDM cohort. Residents aged 18 to 70 years from Houtang Village, Nanyue Town, Yueqing City, Zhejiang Province, China, were invited to participate between October 2018 and July 2019, and subjects with previously diagnosed DM, serious systemic diseases, or receipt of medical therapy that could affect blood glucose (e.g., glucocorticoids) were excluded.

Participation in this study was voluntary, and local residents signed up for the study with their local community doctor. Community doctors selected participants who met the inclusion criteria by inquiring and consulting past medical histories. All participants signed an informed consent form. Ethical approval for this study was obtained from the Ethics Committee of Yueqing People's Hospital, and all examinations were conducted in accordance with the tenets of the World Medical Association's Declaration of Helsinki.

### 2.2. General Study Examinations

Baseline physical examinations were conducted, including the measurement of blood pressure, height, and weight. Each participant's systolic blood pressure (SBP) and diastolic blood pressure (DBP) were measured in sitting position on the right arm using an electronic blood pressure monitor (Omron HEM-7200, China). Two measurements were taken 5 minutes apart, and the mean was used as the final BP reading. Systolic hypertension was defined as SBP ≥140 mmHg and/or diastolic blood pressure ≥90 mmHg. Height was measured to the nearest 0.5 cm using a calibrated stadiometer, and weight was measured to the nearest 0.1 kg in light indoor clothing and bare feet. Waist circumference (WC) was measured to the nearest 0.1 cm at the midpoint between the subcostal margin and the margin of the supracristal plane according to the diagnostic criteria of the International Diabetes Federation (IDF). Hip circumference (HC) was measured to the nearest 0.1 cm around the buttocks, at the height of the greater trochanter, in a standing position. Body mass index (BMI) is calculated as weight in kilograms divided by height in meters squared; BMI >24 was considered as abnormal. The waist-to-hip ratio (WHR) is calculated as waist circumference divided by hip circumference; WHR values of >0.9 and >0.8 were considered to be high for men and women, respectively.

Blood samples were collected from all participants for the measurement of routine blood examination, renal function, electrolytes, fasting plasma glucose (FPG), insulin, lipids, HbA1c, carcino-embryonic antigen (CEA), and alpha fetal protein (AFP) after an overnight fasting of at least 10 hours. All participants underwent a 2-hour 75 g oral glucose tolerance test, and plasma glucose and insulin levels were measured 1 hour (1 h PG) and 2 hours (2 h PG) later. The fasting, 1-hour, and 2-hour venous blood samples were centrifuged and separated immediately and were then transported back to the Yueqing People's Hospital in a blood container at an internal control temperature of 2–10°C within 4 hours of collection. Routine blood measurements were obtained using the hematology analyzer Sysmex XE-2100 (Kobe, Japan), and CEA, AFP, and insulin were measured using an automatic biochemistry analyzer (Roche Cobas E602, Switzerland). HbA1c was measured by high-performance liquid chromatography (HPLC) performed on a Tosoh HLC-723 G8 (Tosoh G8) (Tosoh, Japan). The remaining serum biochemicals were measured using an automatic biochemistry analyzer (Roche Cobas E702, Switzerland).

All baseline participants were screened for DM and PDM according to the American Diabetes Association Standards of Medical Care in Diabetes 2019. Specifically, the diagnostic criteria for DM include (1) FPG ≥7.0 mmol/L, (2) 2-hour plasma glucose ≥11.1 mmol/L during OGTT, or (3) HbA1c ≥6.5%. The diagnostic criteria for PDM include (1) 2-hour plasma glucose ≥7.8 mmol/L and <11.1 mmol/L, (2) FPG ≥5.6 mmol/L and <7.0 mmol/L, or (3) HbA1c between 5.7% and 6.4%.

### 2.3. Questionnaire Interview

The China Chronic Disease and Risk Factor Surveillance Questionnaire (2013) was used for the baseline examination, which included an assessment of sociodemographic and behavioral characteristics via face-to-face interviews. Sociodemographic data included age, gender, education level, and insurance. Behavioral information included smoking, drinking, physical activity, and sleeping time.

Participants were considered to have “vigorous physical activity (PA)” if they performed activities that significantly increased their breath frequency and heart rate for at least 10 minutes at least three times per week. Participants were considered to have “moderate PA” if they performed physical activities that mildly increased their breath frequency and heart rate for at least 10 minutes at least three times per week. Participants were considered to be “inactive in PA” if they performed PA less than 3 times per week for at least 10 minutes.

### 2.4. Statistical Analysis

The data analysis was performed using the commercially available software package Stata 15 (StataCorp, College Station TX, USA). Continuous data were expressed as mean ± standard deviation. One-way ANOVA and chi-squared tests were used to compare the baseline characteristics between participants with newly diagnosed DM and PDM and normal participants. A multinomial logistic regression analysis was used to assess the risk factors for diabetes and prediabetes compared to the nondiabetes group. Variables with a *P* value of  <0.05 during the comparison of the baseline characteristics mentioned above were included in the multiple regression analysis. Fasting insulin, 1 h-PG, 1 h-insulin, 2 h-PG, 2 h-insulin, and the waist-hip ratio were excluded from the multiple regression analysis due to collinearity with other variables. *P* values of less than 0.05 were considered statistically significant.

## 3. Results

There were 604 eligible registered village residents in Houtang Village, Nanyue Town, Yueqing City, in terms of the age requirement, and 436 residents voluntarily participated in this study. Among the 436 village residents who provided informed consent to participate in the study at baseline, we further excluded subjects who reported recently diagnosed DM (*n* = 17) and hepatitis (*n* = 1), as well as those who refused to undergo blood tests (*n* = 7) or the questionnaire interview (*n* = 5). Thus, a total of 406 subjects were included in the baseline analysis. These subjects were classified into three groups based on the FPG and OGTT examination results: newly diagnosed DM (*n* = 58), PDM (*n* = 166), and normal (*n* = 182). The corresponding prevalence of newly diagnosed DM and PDM was 14.3% and 40.9%, respectively.

The mean age of the 406 subjects was 51.2 ± 11.0 years and 160 (33.0%) were men. Significant differences were observed in the distribution of gender, age, and education level between the three groups (*P* < 0.05) (Table 1). The mean age of participants in the DM, PDM, and normal groups was 56.7 ± 7.72, 52.31 ± 11.0, and 48.37 ± 11.02 years, respectively (*P* = 0.001). Among all study participants, 102 (25.1%) were uneducated, including 21 (36.2%) in the DM, 45 (27.1%) in the PDM, and 36 (19.8%) in the normal group. The number of participants with an education level of primary school was 25 (43.1%) in the DM, 58 (34.9%) in the PDM, and 54 (29.7%) in the normal group. The corresponding numbers of participants with an education level of junior high school and college were 12 (20.7%) and 0 (0%), 57 (34.3%) and 6 (3.61%), and 84 (46.2%) and 8 (4.40%) for the DM, PDM, and normal groups, respectively. A total of 370 (91.1%) participants had insurance, including 55 (94.8%) in the DM, 149 (89.8%) in the PDM, and 166 (91.2%) in the normal group. No significant differences in medical insurance were observed among the three groups (*P* = 0.538).

As shown in Table 2, significant differences were observed in the levels of FPG, fasting insulin, 1 h-PG, 1 h-insulin, 2 h-PG, 2 h-insulin, and HbA1c among the three groups (all with *P* < 0.05). Participants with DM had significantly higher blood glucose and insulin levels than those with PDM, and further higher levels than the normal participants.

There were significant differences in the levels of blood lipids, leukocytosis, blood pressure, BMI, and female WHR among the three groups (all with *P* < 0.05) (Table 3). No significant differences were observed in fasting insulin, CR, BUN, CEA, or male WHR (*P* = 0.460, 1.00, 0,339, 0.692, and 0.510, respectively) (Table 3). In addition, AFP levels were within the normal range for all participants.

Regarding behavioral characteristics, there were no significant differences in smoking, alcohol consumption, PA, or sleeping times (all *P* > 0.05) among the three groups (Table 4). The number of smokers in the DM, PDM, and normal group was 12 (20.7%), 33 (19.9%), and 36 (19.8%), respectively. There were 228 drinkers among all 406 participants, including 30 (51.7%) in the DM, 90 (54.2%) in the PDM, and 98 (53.9%) in the normal group. In the DM group, 5 (8.62%) participants reported vigorous PA, while this number was 18 (10.8%) in the PDM group and 18 (9.89%) in the normal group. The number of participants with moderate and inactive PA in the DM, PDM, and normal group was 21 (36.2%), 57 (34.3%), and 64 (35.2%) and 33 (56.9%), 104 (62.7%), and 117 (64.3%), respectively. The average sleeping time was 6.8 ± 1.55 hours in the DM group, 7.17 ± 1.49 hours in the PDM, and 7.00 ± 1.41 hours in the normal group.

Multiple multinomial logistic regression analyses showed that older age (RRR = 1.06; 95% CI, 1.01–1.11, *P* = 0.018), higher systolic blood pressure (RRR = 1.04; 95% CI, 1.004–1.08, *P* = 0.030), higher BMI (RRR = 1.20; 95% CI, 1.06–3.06, *P* = 0.004), higher TG (RRR = 1.80; 95% CI, 1.06–3.06, *P* = 0.029), and higher WBC (RRR = 1.32; 95% CI, 1.07–1.64, *P* = 0.010) were associated with a significantly higher risk of DM (Table 5). Meanwhile, higher systolic blood pressure (RRR = 1.03; 95% CI, 1.004–1.06, *P* = 0.025) was significantly associated with a higher risk of PDM.

## 4. Discussion

Our study reported the prevalence of DM (14.3%) and PDM (40.9%) and the associated factors from the baseline data of a cohort study. We found that older age, higher systolic blood pressure, higher BMI, higher TG, and higher WBC were associated with a higher risk of DM, while only higher systolic blood pressure was associated with a higher risk of PDM.

The prevalence of DM has risen sharply in China in recent years, from 1% in 1980 to 12.8% in 2013 [10, 11]. Meanwhile, the reported prevalence of PDM has also rapidly increased, from 15.5% in 2008 to 35.7% in 2013 [10]. The identified prevalence of DM and PDM in our study was higher than that reported in 2013, suggesting a further increasing trend in the epidemic. A higher prevalence of PDM (50.1%) was reported in another study of Chinese adults [12]. One of the major reasons we conducted the study in Houtang Village of Yueqing City (located in East China, near the coastline) was based on the fast urbanization that happened in China over the past 30 years and our hopes that the data we report here represent the current transition of this chronic disease from urban toward rural areas. Most importantly, this wealthy village exhibits a relative stability of its registered population, which is essential for a cohort study. Discrepancies among the findings could be due to differences in the population characteristics and disease definitions [13].

Consistent with previous studies, subjects with an older age had a higher risk of DM in our study [14–16]. Participants with PDM were also older than the normal participants, but the difference was not statistically significant. This could be due to PDM being milder and more prevalent or simply due to a small sample size. Associations between the education level and the risk of DM and PDM are inconsistent in the literature. We found that participants with DM and PDM had lower education levels compared to the normal participants in our study. This finding was supported by other studies and may be due to people with more education having a better awareness about glycemic control and a higher inclination to maintain a healthy lifestyle [17–19]. In contrast, some studies presented an opposite finding, suggesting that people with a higher education level had a higher DM prevalence, partly due to earlier detection of the disease [20, 21].

In our study, BMI and WHR were higher in the DM and PDM groups compared to the normal group. This finding is consistent with other studies and suggests the role of obesity in the pathogenesis of DM [18, 22–24]. Participants with PDM and DM were more likely to have dyslipidemia compared to the normal participants in our study, and increased TG was found to be a significant risk factor for DM. This finding is supported by Liu et al. who reported that TG was the most prominent factor for the onset of diabetes [25]. Obesity is directly or indirectly associated with myriad metabolic disorders and dysfunctions, including chronic low-grade inflammation and insulin resistance, which are both causally related to the development and progression of DM [26–28]. With an increasing global prevalence, obesity has become a major global public health issue; thus, it is imperative that intervention programs are implemented to control obesity for the prevention of DM.

Similar to previous studies [14, 18, 29, 30], higher systolic blood pressure was found to be associated with a higher risk of both DM and PDM in our study. The pathophysiological mechanisms that explain the association between hypertension and DM include high BP-induced microvascular dysfunction and altered endothelial dysfunction, which have been found to be independent predictors of type 2 diabetes [31]. It is noteworthy that systolic blood pressure was the only risk factor that was associated with both DM and PDM in our study, suggesting its importance in the pathogenesis of DM. Future health-promoting strategies should take the control of systolic blood pressure into consideration for better efficacy.

We found that the WBC count was significantly higher in DM and PDM participants. This finding is supported by Zang et al. who suggested that WBC counts may play an important role in the development of DM or PDM after a six-year study in Chinese adults [32]. A recent meta-analysis that included 90,000 participants also demonstrated a positive correlation between an increased WBC level and DM risk [33]. The WBC count is an indicator of chronic systemic inflammation, suggesting the potential role of inflammation in the development and progression of DM. It has been reported that the prevalence of macro- and microvascular complications increase in a dosage-related manner with the WBC count [34], which may be the underlying reasons for the increased DM risk.

Blood tests for BUN and CR are the simplest way to monitor kidney function and have been used to monitor the progress of diabetic nephropathy [35]. Our study showed that blood CR and BUN levels were not associated with the risk of DM or PDM, while previous studies reported that a higher BUN level and lower CR level were associated with an increased risk of incident DM [35]. Further studies are required to better understand this association and the underlying mechanisms.

It has been reported that smokers are 30 to 40 percent more likely to develop type 2 DM than nonsmokers [36]. However, in our study, smoking was not related to the risk of DM or PDM, which may be because we only assessed newly diagnosed DM and the sample size was relatively small or the self-reported smoking status may have been biased. Studies concerning the effect of alcohol drinking and PA on the development of DM have produced conflicting results. Alcohol drinking and PA were not associated with DM or PDM in our study, which is in line with some previous studies [17, 37]. However, other studies have reporting conflicting results. Zhu et al. suggested that the alcohol drinking status may be valuable in predicting the incidence of diabetes [38], and some observational studies have indicated that moderate alcohol intake demonstrates certain protective effects on the status of DM [39, 40]. The difference in the alcohol-diabetes relationship may be attributable in part to disparities in the gender distribution of different studies [41] and an artifact of referent group selection, particularly when the confounder adjustment is weak [42]. It has also been suggested that PA is useful in preventing or delaying the onset of type 2 DM [40, 43], by improving insulin sensitivity and assisting in reducing elevated blood glucose levels to the normal range [41].

The limitations of this study need to be noted. First, this was not a population-based study and the sample size was relatively small, hindering the generalizability of the study findings. Second, the study was cross-sectional in design and thus could not prove causal relationships between the risk factors and DM or PDM. Third, the data on smoking, alcohol drinking, and PA were self-reported and may suffer from recall bias, and how this may bias the study findings is unknown. Despite these limitations, we included multiple sources of potential risk factors, including sociodemographic, biochemical, anthropometric, and behavioral characteristics, which enabled a comprehensive risk assessment for DM and PDM.

## 5. Conclusions

In conclusion, we reported the prevalence of newly diagnosed DM and PDM and the associated risk factors based on the baseline data of our study cohort from a wealthy village in East China. Longitudinal data are needed to better understand the etiology of PDM onset and its progression to DM, so that we can explore the potential intervention methods to delay or reverse this transformation.

## Figures and Tables

**Table 1:** Baseline sociodemographic characteristics of study participants.

Parameters	Total (*n* = 406)	Newly diagnosed DM (*n* = 58)	PDM (*n* = 166)	Normal (*n* = 182)	*P* value
Male n(%)	160 (33.0)	28 (48.3)	72 (43.4)	60 (33.0)	0.046
Age (years)	51.2 (11.0)	56.77 ± 7.72	52.31 ± 11.0	48.37 ± 11.02	<0.001
*Education n(%)*					0.005
Uneducated	102 (25.1)	21 (36.2)	45 (27.1)	36 (19.8)	—
≤ Primary school	137 (33.7)	25 (43.1)	58 (34.9)	54 (29.7)	—
Junior high school	153 (37.7)	12 (20.7)	57 (34.3)	84 (46.2)	—
≥ College	14 (3.45)	0 (0.00)	6 (3.61)	8 (4.40)	—
Insurance n(%)	370 (91.1)	55 (94.8)	149 (89.8)	166 (91.2)	0.538

DM = diabetes mellitus and PDM = prediabetes mellitus.

**Table 2:** Baseline FPG, insulin, and HbA1c levels of study participants.

Parameters	Newly diagnosed DM (*n* = 58)	PDM (*n* = 166)	Normal (*n* = 182)	*P* value
FPG (mmol/L)	6.68 ± 1.28	5.52 ± 0.63	4.97 ± 0.34	<0.001
Fasting insulin (uU/ml)	9.74 ± 5.64	9.39 ± 7.86	7.27 ± 4.72	0.002
1 h-PG (mmol/L)	14.85 ± 2.76	10.69 ± 2.15	8.37 ± 2.10	<0.001
1 h-insulin (uU/ml)	54.91 ± 33.05	80.07 ± 65.67	78.13 ± 68.21	0.027
2 h-PG (mmol/L)	14.10 ± 2.80	8.54 ± 1.44	6.14 ± 0.93	<0.001
2 h-insulin (uU/ml)	74.98 ± 55.38	86.52 ± 76.03	55.36 ± 41.92	<0.001
HbA1c (%)	6.39 ± 0.83	5.72 ± 0.69	5.42 ± 0.36	<0.001

DM = diabetes mellitus, PDM = prediabetes mellitus, FPG = fasting plasma glucose, 1 h-PG = 1-hour plasma glucose, 2 h-PG = 2-hour plasma glucose, and HbA1c = hemoglobin A1C.

**Table 3:** Baseline biochemical and anthropometric measurements of study participants^*∗*^.

Parameters	Newly diagnosed DM (*n* = 58)	PDM (*n* = 166)	Normal (*n* = 182)	*P* value
Fasting insulin ≥25 (uU/ml)	1 (1.72)	3 (1.81)	1 (0.55)	0.460
TG > 1.70 (mmol/L)	33 (56.9)	72 (43.4)	42 (23.1)	<0.001
TC > 5.70 (mmol/L)	27 (46.6)	58 (34.9)	46 (25.3)	0.007
HDL-C < 1.00 (mmol/L)	1 (1.72)	15 (9.04)	4 (2.20)	0.009
LDL-C > 3.10 (mmol/L)	37 (63.8)	115 (69.3)	82 (45.1)	<0.001
WBC > 10.0 (×10^9^/L)	4 (6.90)	6 (3.61)	2 (1.10)	0.049
CR > 104 for male and >84 for female (umol/L)	0 (0.00)	0 (0.00)	1 (0.55)	1.000
BUN > 7.20 (mmol/L)	6 (10.3)	11 (6.63)	9 (4.95)	0.339
AFP > 13.4 (ng/ml)	0 (0.00)	0 (0.00)	0 (0.00)	—
CEA > 5.00 (ng/ml)	1 (1.72)	8 (4.82)	7 (3.85)	0.692
Systolic blood pressure (≥140 mmHg)	39 (67.2)	89 (54.3)	58 (31.9)	<0.001
Diastolic blood pressure (≥90 mmHg)	20 (34.5)	45 (27.4)	31 (17.0)	0.009
Body mass index (≥24)	40 (69.0)	95 (57.6)	69 (37.9)	<0.001
Waist-hip ratio	47 (81.0)	133 (80.6)	131 (72.0)	0.117
Male >0.9	19 (67.9)	43 (60.6)	33 (55.0)	0.510
Female >0.8	28 (93.3)	90 (95.7)	98 (80.3)	0.002

^*∗*^Data are expressed as number (percentage). DM = diabetes mellitus, PDM = prediabetes mellitus, TG = triglycerides, TC = total cholesterol, HDL-C = high-density lipoprotein cholesterol, LDL-C = low-density lipoprotein cholesterol; WBC = white blood cell, CR = creatinine, BUN = blood urea nitrogen, CEA = carcino-embryonic antigen, and AFP = alpha fetal protein.

**Table 4:** Baseline behavioral characteristics of study participants.

Parameters	Newly diagnosed DM (*n* = 58)	PDM (*n* = 166)	Normal (*n* = 182)	*P* value
*Smoking* ^*∗*^				0.988
Nonsmoker	46 (79.3)	133 (80.1)	146 (80.2)	
Smoker	12 (20.7)	33 (19.9)	36 (19.8)	
*Alcohol consumption* ^*∗*^				0.946
Nondrinker	28 (48.3)	76 (45.8)	84 (46.2)	
Drinker	30 (51.7)	90 (54.2)	98 (53.9)	
*Physical activity* ^*∗*^				
Vigorous	5 (8.62)	18 (10.8)	18 (9.89)	0.883
Moderate	21 (36.2)	57 (34.3)	64 (35.2)	0.965
Inactive	33 (56.9)	104 (62.7)	117 (64.3)	0.599
Sleeping time (hours)	6.8 (1.55)	7.17 (1.49)	7.00 (1.41)	0.230

^*∗*^Data are expressed as number (percentage). DM = diabetes mellitus, and PDM = prediabetes mellitus.

**Table 5:** Baseline multiple multinomial logistic regression analysis of risk factors for diabetes and prediabetes compared to the nondiabetes group in study participants.

Variables	Newly diagnosed DM vs. normal		PDM vs. normal	
	RRR (95% CI)	*P* value	RRR (95% CI)	*P* value
Age (years)	1.06 (1.01, 1.11)	0.018	1.02 (0.99, 1.05)	0.140
Male	0.73 (0.34, 1.55)	0.412	0.97 (0.58, 1.64)	0.922
Junior high school or above	0.43 (0.17, 1.06)	0.068	0.74 (0.41, 1.32)	0.310
Systolic blood pressure (mmHg)	1.04 (1.004, 1.08)	0.030	1.03 (1.004, 1.06)	0.025
Diastolic blood pressure (mmHg)	1.00 (0.98, 1.02)	0.805	1.01 (0.99, 1.02)	0.488
Body mass index (>24 kg/m^2^)	1.20 (1.06, 1.35)	0.004	1.07 (0.98, 1.16)	0.151
TG (mmol/L)	1.80 (1.06, 3.06)	0.029	1.40 (0.92, 2.14)	0.116
TC (mmol/L)	1.12 (0.31, 4.06)	0.860	0.90 (0.34, 2.42)	0.836
HDL-C (mmol/L)	2.93 (0.60, 14.4)	0.186	0.95 (0.29, 3.12)	0.936
LDL-C (mmol/L)	1.31 (0.37, 4.62)	0.671	1.51 (0.57, 3.99)	0.405
WBC (×10^9^/L)	1.32 (1.07, 1.64)	0.010	1.17 (1.00, 1.38)	0.052

DM = diabetes mellitus, PDM = prediabetes mellitus, RRR = relative risk ratio, TG = triglycerides, TC = total cholesterol, HDL-C = high-density lipoprotein cholesterol, LDL-C = low-density lipoprotein cholesterol; WBC = white blood cell, RRR = relative risk ratio, and CI = confidence interval.

## Data Availability

All data used during the study are available from the corresponding author by request.
